# Strain-level genomic analysis of *Staphylococcus epidermidis* across multiple body sites in healthy females

**DOI:** 10.1128/spectrum.00114-26

**Published:** 2026-06-22

**Authors:** Sandra Jablonska, Niru Shanbhag, Alex Kula, Catherine Putonti

**Affiliations:** 1 Bioinformatics Program, Loyola University Chicago 2456, Chicago, Illinois, USA; 2 Department of Biology, Loyola University Chicago 2456, Chicago, Illinois, USA; Universidad Nacional Autonoma de Mexico - Campus Morelos, Cuernavaca, Mexico

**Keywords:** *Staphylococcus epidermidis*, healthy female microbiome, urinary microbiome, skin microbiome, oral microbiome, nasal microbiome

## Abstract

*Staphylococcus epidermidis* is a ubiquitous skin commensal that also colonizes the nasal and oral cavities, as well as the gastrointestinal and urinary tracts. Although prior studies have found genetic variation between *S. epidermidis* from infections and commensal strains, prior studies also have found that individual strains can inhabit multiple sites within an individual. None of these studies, however, have considered urogenital isolates. Here, we collected samples from 76 healthy female participants and performed whole-genome sequencing on 114 *S. epidermidis* isolates from the skin, nasal cavity, oral cavity, and urine. Pairwise average nucleotide identity (ANI) revealed instances of identical strains (>99.99% ANI) across multiple body sites within individuals and, in some cases, between individuals. Accessory genome functional profiles showed no clustering by anatomical site, indicating limited niche specialization and a broadly shared accessory gene pool. Intact prophages were shared among isolates, consistent with a fluid, mobile accessory genome. Although multinomial logistic regression using gene-cluster presence/absence identified site-associated gene enrichment, the model achieved low accuracy in predicting isolation source from publicly available genomes. The absence of strong evidence for niche-specific adaptation among isolates from healthy individuals supports the species’ generalist lifestyle and genetic diversity. This study advances our understanding of *S. epidermidis* population structure in healthy hosts, including the urinary tract.

**IMPORTANCE:**

While traditionally considered a benign skin colonizer, *Staphylococcus epidermidis* is also a resident and transient member of the nasal, oral, gastrointestinal, and urinary microbiota. Strain-level diversity and ecological adaptations in healthy humans remain underexplored. This study reveals that the same *S. epidermidis* strains can colonize multiple body sites in the same individual, highlighting a generalist colonization strategy. This is further supported by our development of a machine-learning model, which has a relatively low accuracy in predicting the isolation source for strains that are not associated with infections. This study provides a genomic framework for distinguishing commensal adaptation from pathogenic potential.

## INTRODUCTION

*Staphylococcus epidermidis* primarily functions as a commensal organism on epithelial surfaces such as skin, nasal passages, and mucosal linings, where it contributes to the maintenance of the epidermis, protection against pathogenic organisms, and modulation of host immune responses (1). A study by Garcia-Gutierrez et al. (2) compared *S. epidermidis* strains isolated from fecal and skin samples, revealing interactions within stress response, biofilm formation, and metabolism. These findings suggest ecological flexibility in *S. epidermidis*, supporting its ability to colonize diverse body sites. Furthermore, genomic analyses have identified evidence of the same *S. epidermidis* strain at different anatomical sites within the same individual (3, 4). Similar observations have been made for other staphylococci of the human body (5).

Comparative genomic analyses of *S. epidermidis* have shown that it possesses an open pangenome, with up to 20% of the genome in flux (6, 7). These accessory genes include genes associated with antibiotic resistance and adhesion (8). In the pangenome analysis by Su et al. (7), 25 genes associated with biofilm formation and cell toxicity were more frequently found in the genomes of skin and blood isolates than in isolates from other sources, including both other anatomical sites and environments. *S. epidermidis* from the skin can cause implant-associated infections, prosthetic valve endocarditis, and cardiac pacemaker infections (9). Moreover, nosocomial bloodstream *S. epidermidis* infections can arise from long-term catheter use (10). Prior research suggests that an *S. epidermidis* isolate’s ecology can be determined based on genomic content, identifying six genetic clusters, based on multi-locus sequence typing (MLST), within the species (11). In this and other similar studies, the focus was on distinguishing between hospital and nonhospital sources (11–13).

To investigate whether *S. epidermidis* strains from different anatomical sites represent distinct lineages or share common origins, we performed whole-genome sequencing of isolates collected from the oral cavity, nasal cavity, skin, and urine of 76 healthy female participants. In contrast to the skin and oro-nasal cavity, the urinary microbiota is understudied (14). While a higher incidence of *S. epidermidis* has been detected in urinary samples of females with stress urinary incontinence (15), its role (if any) in the urinary tract has not been thoroughly investigated. Machine learning was used to identify genes within the genomes of these commensal isolates to explore site-specific adaptations. This multilayered genomic approach is a critical first step in understanding the population structure, ecological plasticity, and evolutionary strategies of *S. epidermidis* in the healthy human host.

## RESULTS

### *S. epidermidis* of the oral, nasal, skin, and urinary tract

Oral swabs, nasal swabs, skin swabs, and midstream urine samples were collected from 76 females (18 ≤ age ≤ 25) as part of an IRB-approved study (see Materials and Methods). Participants represented asymptomatic “healthy” individuals who had not taken antibiotics in the past 6 months. In total, 304 samples were collected from the 76 participants. Using selective media (see Materials and Methods), *S. epidermidis* was successfully isolated and sequenced from 10 oral, 41 nasal, 34 skin, and 29 urine samples (Fig. S1). Species confirmation was conducted by whole-genome sequencing. Genomes ranged in size from 2.38 Mbp to 2.65 Mbp. Details about the *S. epidermidis* genomes produced from these samples are included in Table S1.

MLST analysis of the 114 *S. epidermidis* genomes identified 41 distinct sequence types (STs). Fifteen genomes could not be assigned (nasal = 7, skin = 3, urine = 5). ST73 was the most common ST among the genomes (*n* = 20) and was exclusive to oral, nasal, and skin isolates. While a statistically significant association between isolation source and ST was identified (chi-squared test, *P* = 0.0004), pairwise comparisons between isolation sources were not statistically significant, a likely effect of low-frequency STs. There are seven unique STs among the oral isolates, 21 among the nasal isolates, 15 among the skin isolates, and 17 among the urine isolates. Post-hoc Pearson residuals were computed, identifying that nasal isolates were more frequently ST35 and oral isolates were more frequently ST59 than expected. STs for each of the *S. epidermidis* genomes are listed in Table S1.

**Fig 1:**
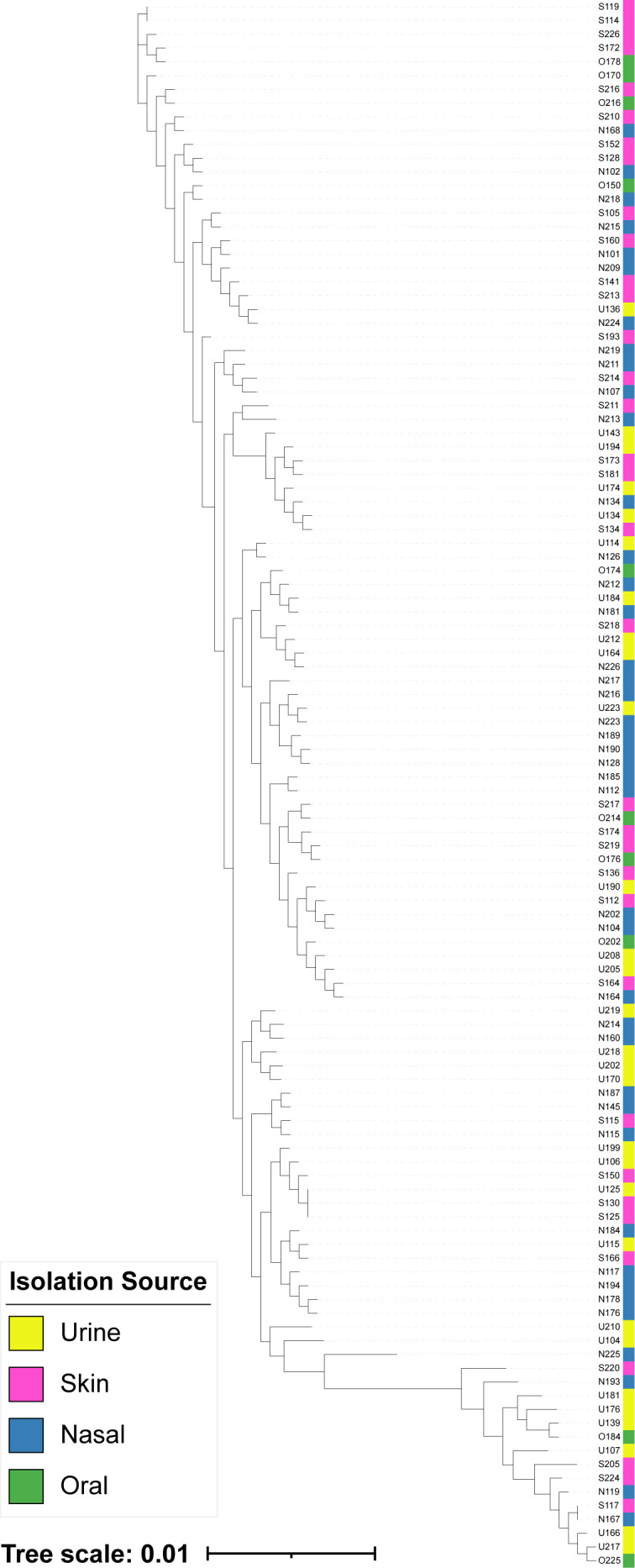
*S. epidermidis* core phylogenetic tree of all isolates collected from participants. The core genome includes single copy number genes identified in all genomes examined. Each tip represents an individual isolate’s genome labeled acorrding to the anatomical origin and participant ID assigned. Colored bars denote the anatomical origin of each isolate (e.g., oral cavity, nasal cavity, skin, urine).

Whole-genome sequences of the 114 isolates were next compared to each other to assess sequence similarity. A total of 4,875 unique gene clusters (homologous genes) were identified across the 114 genomes, representing the pangenome for these isolates. The single copy number core genome comprises 832 genes. These single copy core genes were identified using an MCL inflation parameter of 10, the most sensitive value possible and recommended when analyzing strains of the same species; the selected stringency of our method for identifying the “core genome” focuses our analysis on only highly conserved gene sequences. A phylogenomic tree constructed from this core genome tree shows that genomes from isolates collected from the same source (oral, nasal, skin, or urine) did not form a single clade (Fig. 1). However, we identified instances in which genomes from the same participant had a core genome more similar to one another than to any other genome under consideration. Despite these few instances, this analysis suggests that the core genome does not provide sufficient resolution to reliably infer the anatomical site of isolation for *S. epidermidis* strains.

**Fig 2:**
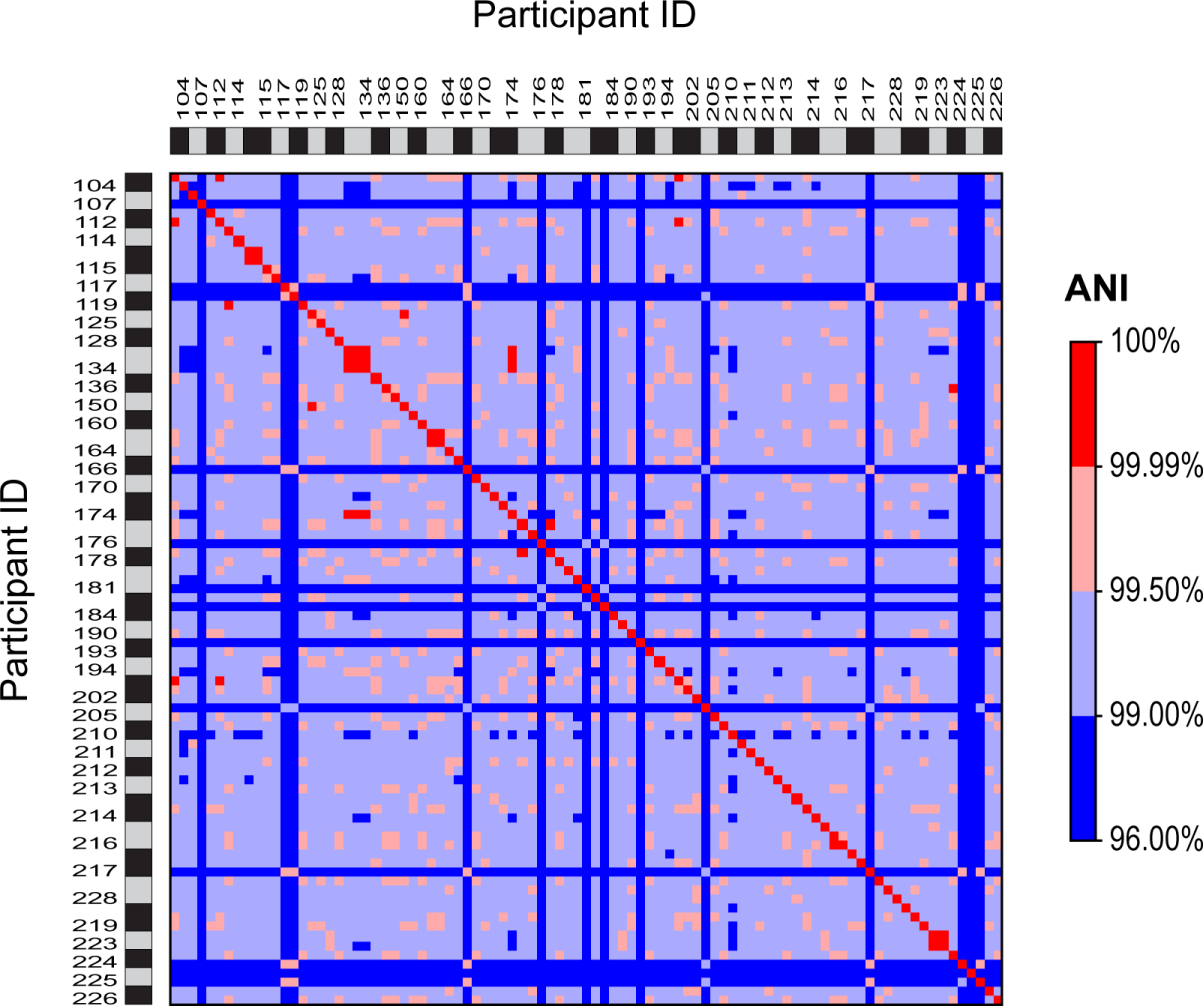
Heatmap of pairwise ANI values calculated between 91 of the 114 *S. epidermidis* genomes included in this study. Each cell represents the ANI value between two genomes, with values shown as a color gradient from 96% (dark blue) to 100% (red); all ANI values exceeding the strain threshold of 99.99% are shown in red. (Note, the minimum pairwise ANI value is 96.53%.) Genomes are organized by participant, and the outer gray and black bands indicate that the genomes are from the same participant.

Next, pairwise average nucleotide identity (ANI) comparisons of the 114 genomes were performed (Table S2). Pairwise ANI values ranged from 96.5304% (N225 and S111) to 99.9996% (N104 and S112). Narrowing our focus to only genomes from participants in which more than one sample produced a *S. epidermidis* genome (*n* = 91; Fig. 2), we investigated if the same strain resided in different anatomical sites. We used the ANI > 99.99% threshold, previously suggested by others (16), to identify genomes likely representative of the same strain. Participants 115 and 164 had nasal and skin isolates exceeding this threshold, with pairwise ANI values of 99.9960% and 99.9985%, respectively. The genomes from the oral and skin samples of participant 216 exceeded this threshold (99.9929%) as did the nasal and urine samples of participant 223 (99.9995%). In participant 135, the genomes from the nasal, skin, and urine samples had an average pairwise ANI value of 99.9967%. We also identified genomes from the same isolation source, but from different participants, that exceeded the ANI strain threshold, namely, the skin isolates from participant 125 and 150 (99.9995%), the nasal isolates from participants 104 and 202 (99.9989%), and the urine isolates from participants 134 and 174 (99.9954%).

### *S. epidermidis* accessory genome

**Fig 3:**
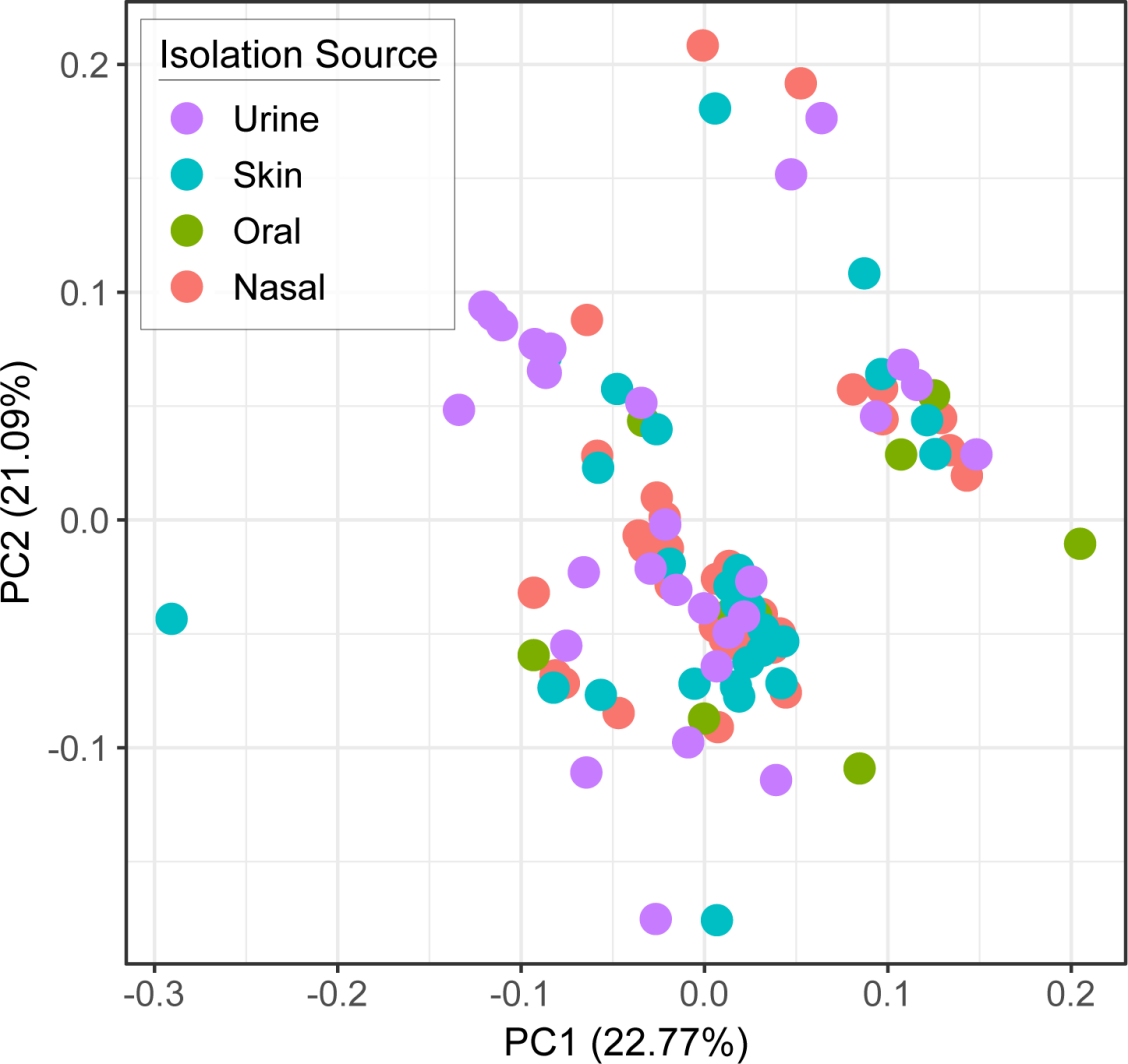
Principal coordinate analysis based on a Jaccard dissimilarity matrix generated from the presence/absence of COGs within the accessory genomes of the 114 *S. epidermidis* genomes. Each point represents a genome, colored by anatomical site of isolation (oral, nasal, skin, urine).

Given that the core genome showed no sequence variation associated with the ecological niche, we next examined the accessory genome. Using COG20 functional annotations, we examined functionality within the accessory genome; here, COGs were considered accessory if they were absent from at least one genome. In total, we identified 148 COGs. As illustrated in Fig. 3, the genomes do not cluster by isolation source based on the functions encoded in their accessory genome. Furthermore, statistical analyses revealed that no accessory COGs were significantly associated with a particular anatomical site (PERMANOVA, *P* = 0.203; *R*^2^ = 0.033).

**Fig 4:**
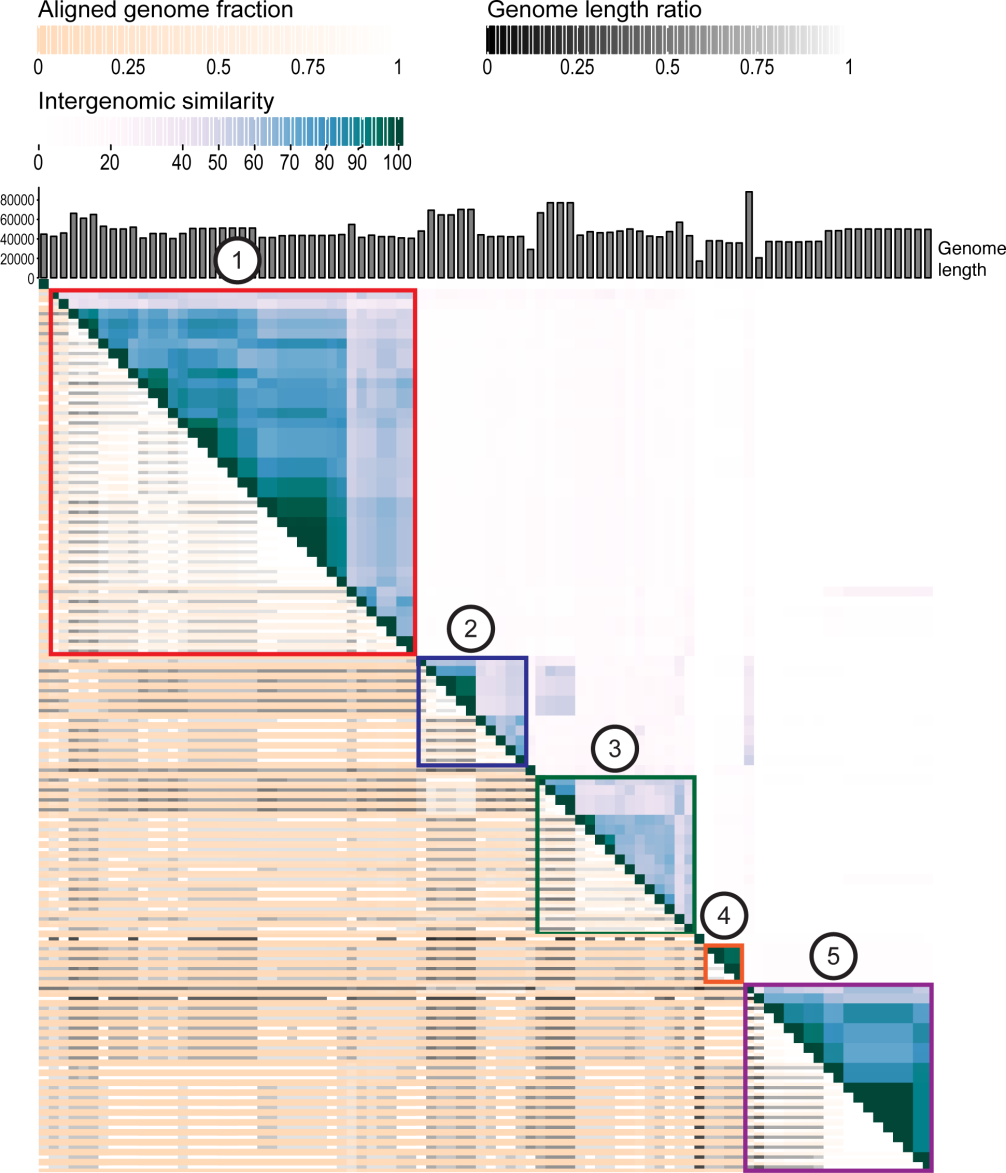
VIRIDIC computed pairwise intergenomic similarities of PHASTEST identified sequences. The upper triangle represents pairwise average nucleotide identity (ANI), with darker shades (green to blue) indicating higher sequence similarity. The lower triangle represents the fraction of the genomes aligned for calculating the ANI value, with lighter colors indicating a larger portion of the genome included. Five manually annotated clusters, labeled 1–5, highlight key regions with high intergenomic similarity (>99.9%). Prophages outside these five blocks mostly show low intergenomic similarity to the clustered groups and to one another, suggesting greater sequence divergence.

Although no overall association between accessory gene functionality and isolation source was observed, we decided to further investigate specific classes of accessory genes, namely, prophages, antibiotic resistance genes (ARGs), and virulence factors. Out of the 114 *S. epidermidis* genomes analyzed, 104 (91.23%) of the genomes contained at least one predicted prophage sequence. In total, 90 intact, 11 questionable, and 75 incomplete prophage sequences were identified. Focusing on the predicted intact prophages, 69 genomes harbored 1 intact prophage sequence, 9 genomes included 2, and 1 genome included 3 prophage sequences. The 90 intact prophages represented 5 distinct clusters of related phage sequences as well as prophage sequences that are unique among the set examined (Fig. 4). VIRIDIC analysis determined that these intact prophages represent 32 unique genera. The genus with the most members (*n* = 17) included representatives from all four sites sampled, while the next two largest genera only included prophages from genomes of isolates from nasal, skin, and oral samples (Table S3). By comparing the prophage sequences to characterized sequences, we found that all five of the clusters were most similar to characterized phages known to infect members of the Bacillota phylum (formerly Firmicutes) (Fig. S2).

We next considered prophages as a marker of shared evolutionary history. One could hypothesize that *S. epidermidis* strains that derived from the same ancestor could have the same prophage sequence. Referring to the five participants with highly similar genome isolates, intact prophage sequences were compared; intact prophage sequences were not identified in the 2 genomes from participant 216. Table 1 lists the genomic similarity measured for the prophages in the other four participants. While the prophage sequences of N223 and U223, S134 and U134, and N164 and S164 are identical (100%), some variation is observed for N115 and S115 as well as N134 and S134 and U134. As previously discussed, the genomes N164 and S164 were predicted to be representative of the same *S. epidermidis* strain, with U164 representative of a different strain. Similarly, U164 harbors a different intact prophage. Returning to our analysis of all predicted intact prophage sequences (Table S3), we find that the phage species in the participant 134 and 164 genomes are also found in other genomes in our study; thus, we cannot rule out horizontal rather than vertical acquisition.

**TABLE 1:** Sequence similarity comparison (%) of prophages found in the genomes from participants in which genomes representative of the same strain were identified by ANI

	Sample genome ID		
Participant 115	N115	S115	
N115	100	99.5	
S115		100	
Participant 134	N134	S134	U134
N134	100	95.9	95.9
S134		100	100
U134			100
Participant 164	N164	S164	U164
N164	100	100	1.2
S164		100	1.2
U164			100
Participant 223	N223	U223	
N223	100	100	
U223		100	

Every genome analyzed contained at least one gene associated with antibiotic resistance (ARGs). Three genes (*dfrC*, *norA*, and *mgrA*) were identified in all 114 genomes. Table S4 lists each of the ARGs identified and the number of genomes encoding for the gene for each of the four isolation sources. The PC1 β-lactamase gene *blaZ* was also highly prevalent, detected in 91 (81%) genomes. There is no statistically significant association between *blaZ* presence/absence and isolation source (Fisher–Freeman–Halton exact test; *P* = 0.379). In fact, none of the ARGs were more frequently identified in genomes for an isolation source. Only 3 virulence-associated genes were identified in the 114 *S. epidermidis* genomes examined, all encoding adhesion-related surface proteins. *clfA* was detected in a single isolate (U136), *sdrC* in a single isolate (U106), and *sdrD* in two isolates (U136 and S117).

### Identifying genes associated with strains from the same anatomical site

A multinomial logistic regression (MLR) model using gene presence/absence data and isolation source was derived from 104 of our participant-derived genomes; genomes from the oral cavity were removed from consideration as we only had 10 representatives within our set. The resulting MLR model was then tested on 108 publicly available genomes, including *S. epidermidis* genomes from skin (*n* = 33), urine (*n* = 37), and nasal (*n* = 38) samples. The model achieved a training accuracy of 71.15%, correctly classifying 74 out of 104 genomes. The testing accuracy dropped to 36.11%, with 39 out of 108 genomes correctly predicted. On completely unseen data, our model performed slightly better than chance.

**Fig 5:**
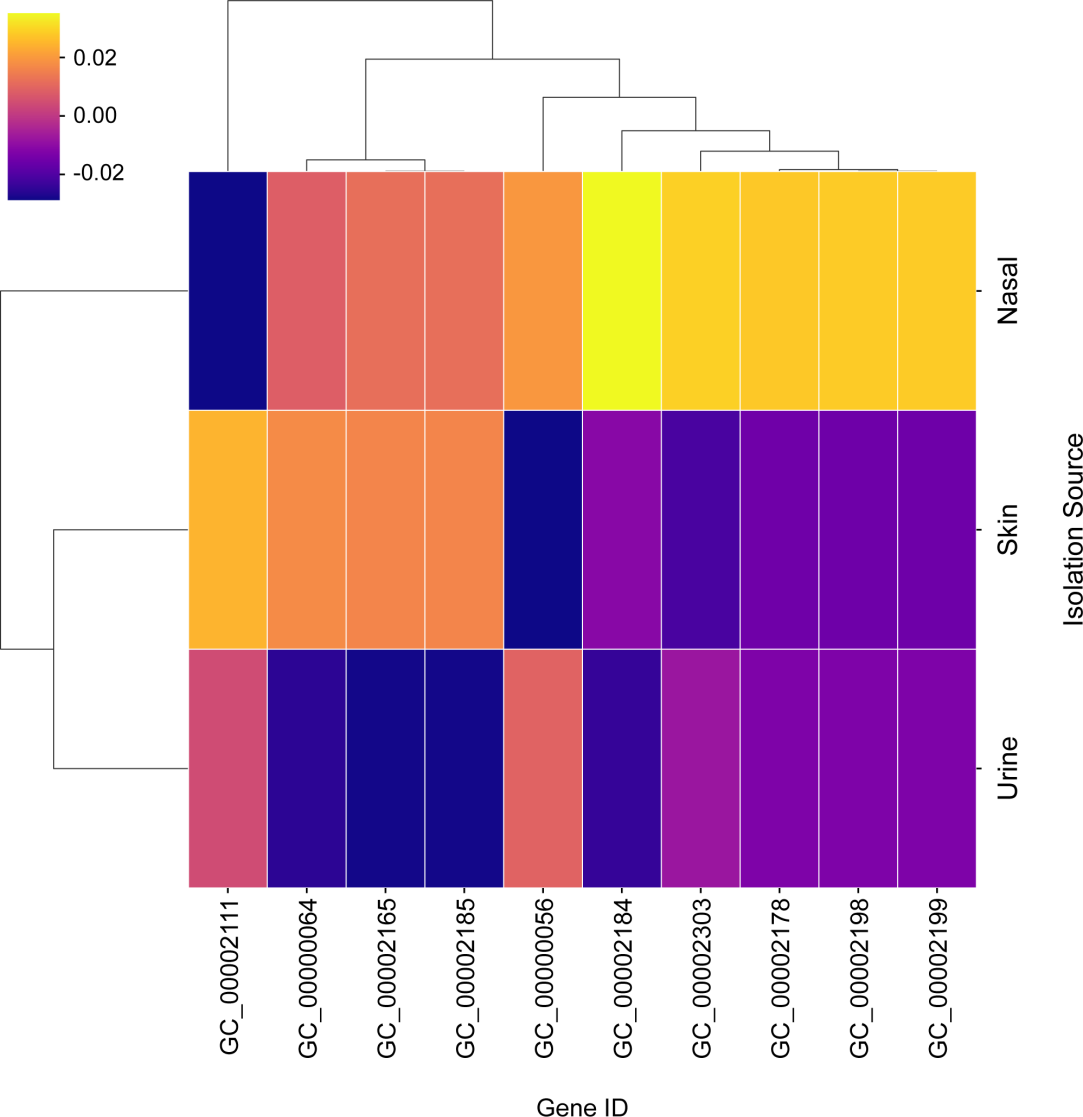
Coefficient heatmap of the top ridge-regularized multinomial features used to differentiate *S. epidermidis* isolates from three anatomical sites: nasal, skin, and urine. Top logistic regression features were chosen if the absolute value of a coefficient was greater than 0.025 in at least one class. A breakdown of the fitted model, including the estimated coefficients, is presented. Columns represent homologous gene IDs (Anvi’o ID numbers assigned during construction of the pangenome), and rows represent body sites of isolation. The color scale indicates the logistic regression coefficient associated with each gene’s contribution to niche classification (yellow = positive, purple = negative).

Genes enriched by anatomical site showed both overlap and divergence in functional profiles. We focused on genes with coefficients greater than 0.025 in at least one isolation source (Fig. 5). Here, we refer to the identified genes by their gene ID number (prefix “GC_”), which were assigned as part of our core genome analysis; the functionality associated with each gene is listed in Table 2. The presence of GC_00000064, GC_00002111, GC_00002165, and GC_00002185 was associated with an increased odds of belonging to the skin class (i.e., genomes from skin isolates). The presence of GC_00000056, GC_00002178, GC_00002184, GC_00002198, GC_00002199, and GC_00002303 was associated with an increased odds of belonging to the nasal class. While two genes in genomes from urine isolates (GC_00000056 and GC_00002111) had positive coefficients, the nasal and skin classes for these genes had higher values. Thus, we did not report any genes in Table 2 for the urine class.

**TABLE 2:** Key genes and associated class coefficients from the multinomial logistic regression model trained on participant samples^*a*^

Isolation site	Gene ID	Coefficient value	COG function/category
Skin	GC_00000064	0.017187	Recombinase
	GC_00002111	0.024526	Uncharacterized protein
	GC_00002165	0.016248	Cell wall/membrane/envelope biogenesis; N-acetylmuramoyl-L-alanine amidase CwlA (CwlA)
	GC_00002185	0.016248	Function unknown; Uncharacterized membrane protein
Nasal	GC_00000056	0.019435	Uncharacterized conserved protein; DUF1643 domain
	GC_00002178	0.02776	ATP synthase associated protein
	GC_00002184	0.035169	Uncharacterized protein
	GC_00002198	0.028252	Transcription, DNA-binding transcriptional regulator, LysR family (LysR) (PDB:2FYI)
	GC_00002199	0.028252	Carbohydrate transport and metabolism; Predicted arabinose efflux permease AraJ, MFS family (AraJ) (PDB:4LDS)
	GC_00002303	0.029083	tRNA ligase

^*a*^ Key genes are defined those with at least one coefficient with an absolute value of 0.025 or greater out of the three classes (isolation sources). The class of the key gene/coefficient and the associated COG function of the key gene are also listed.

## DISCUSSION

Here, we evaluated *S. epidermidis* strains collected from the oral, nasal, skin, and urinary microbiota of healthy females. The inability to isolate *S. epidermidis* from a given sample does not necessarily mean that it was not present in the sample. Instead, it may reflect (i) competition from other *Staphylococcus* species or unrelated genera on the selective media, (ii) low relative abundance of *S. epidermidis* in the sample, or (iii) sampling error. Other staphylococci were identified in these samples, including *S. aureus*, *S. capitis*, *S. haemolyticus*, and *S. hominis* (17–21). Nevertheless, *S. epidermidis* was retrieved from healthy individuals from all four sites.

From MLST analysis alone (Table S1), we can see that each isolation site is inhabited by *S. epidermidis* representing several different STs. Prior studies have found that an individual’s skin (4, 22) and nasal cavity (3) can harbor multiple different STs, and ST diversity within the nares was greater among healthy individuals than patients (23). Among our genomes, three STs were found in samples from different anatomical sites. ST73, ST329, and ST487 were represented by isolates from oral, nasal, and skin samples; these latter two also were represented in urine isolates. However, MLST provides only a high-level overview of the genetic diversity within these isolates. While prior studies have found the same bacterial species within nasal, skin, and oral microbiota, the specific strains colonizing each site often differ (22, 24, 25). While generally true, we found instances in which highly similar or near-identical strains were present across different anatomical sites within the same individual. This observation aligns with previous studies that have detected the same bacterial strain at different anatomical sites within the same individual (26–28).

Because of the environmental and physiological differences between the oral, nasal, and skin environments and the urinary tract, we hypothesized that urinary isolates would be more distinct. This hypothesis emerged from previous microbiome studies that found urinary microbiota to be taxonomically separate from both gut (29) and vaginal communities (26). While largely true, our results identified that the genomes from participant 134’s nasal, skin, and urine isolates exceeded the ANI strain threshold of 99.99%. Prophage analysis further suggests that these isolates derived from a common ancestor: S134, U134, and N134 are all representatives of the same species (Table 1; Table S3). However, because this same phage species was also found in U174, we cannot rule out that the three isolates from participant 134 acquired this prophage independently. Collectively, these findings suggest that individual *S. epidermidis* strains are capable of successfully colonizing diverse anatomical sites within an individual, even when local environmental pressures differ substantially.

Beyond intra-individual comparisons, we also identified instances of highly similar strains isolated from the same anatomical site across different participants. This suggests clonal *S. epidermidis* lineages circulating within the broader population, potentially facilitated by shared environments or frequent interpersonal transmission. Although these genomes are from different samples from different participants, it is unknown if these individuals were in contact with each other. Within-household transmission of microbes has been observed within the gut and oral microbiomes (30). This includes evidence of the same strain shared among cohabitants and among individuals in the same population (31). Thus, the observed evidence of the same strain in different individuals could be that they were roommates, friends, coworkers, or just simply members of the same population (as all participants were students at Loyola University Chicago).

Analysis of the functionality encoded by the accessory genome of the 114 genomes produced here revealed no significant clustering by anatomical site (Fig. 3). No COGs were significantly associated with any anatomical site, suggesting high gene flow and limited niche specialization among *S. epidermidis* isolates, as previously suggested (7, 32). Encoded within this accessory genome are mobile genetic elements, including phages, as well as virulence and antibiotic resistance genes. While prophages are common in *S. epidermidis* genomes (33), they often do not encode for virulence genes. This is in stark contrast to *S. aureus* whose prophages have routinely been associated with pathogenicity in clinical isolates (34). However, *S. epidermidis* phages can transduce antimicrobial resistance plasmids and genomic islands (35). Prior studies have attributed mobile genetic elements to shaping both *S. epidermidis’* genomic diversity and adaptability (9, 36, 37).

Recent analyses of *S. aureus* genomes have shown that shared prophage content, in combination with core genome phylogenomic signals, is consistent with recent common ancestry (38, 39). Concordance among core genome (Fig. 1), ANI (Fig. 2), and prophage sequence similarity (Table 1) suggests that *S. epidermidis* strains within each participant share a recent common ancestor. Interestingly, we identified many of the same prophage species across isolates from different anatomical sites and individuals (Table S3). While a recent review explored the genetic diversity of *S. epidermidis* infecting phages, this analysis was limited to 60 phages with deposited genome sequences (40). To the best of our knowledge, no comprehensive investigation of *S. epidermidis* temperate phages has been conducted.

Patterns of antibiotic-resistance gene diversity differed across anatomical sites, suggesting that local ecological pressures and/or microenvironmental conditions shape the accessory resistome of *S. epidermidis*. Nasal, skin, and urinary genomes had a broader ARG repertoire than the genomes of oral isolates. The nares has a documented role as a reservoir for mobile genetic elements and horizontally acquired resistance (41, 42), and the skin surface is a hotspot for strong environmental selection pressures and gene exchange (6, 22, 43). Fewer ARGs in the genomes of oral isolates could reflect competitive filtering or differential antibiotic exposures at this site (44, 45). For instance, *mecA* was not detected in any of the 10 oral genomes; however, it was identified in 5 nasal, 4 skin, and 5 urine isolate genomes. Similarly, tetracycline resistance genes were not identified in the oral genomes but were found in genomes from the other three sites.

Through machine-learning analysis, we found that the presence/absence of specific genes was associated with *S. epidermidis* strains isolated from specific sites. For instance, *atlE* has an increased odds of being encoded by genomes isolated from skin samples (1.016×). Prior research has found associations between *S. epidermidis* AtlE and primary attachment to polystyrene surfaces (46) and biofilm matrix formation (47). Gene IDs GC_00002198 and GC_00002199, both of which have an increased odds of presence in genomes from nasal swabs, also have been implicated in virulence and biofilm formation (48, 49). Overall, genes enriched in one anatomical site (indicated in Fig. 5 in yellow) are frequently not, or not as, enriched in another. These findings align with broader microbial adaptation theories, where specific gene acquisitions or retentions enable survival under selective pressures (50, 51). Furthermore, these observations support the hypothesis that *S. epidermidis* undergoes extensive strain mixing and exhibits weak niche-specific population structure across healthy body sites (6, 52).

Three prior studies have attempted to use genomic variation to predict the source of isolation of *S. epidermidis* (11–13). More specifically, these studies have focused on distinguishing between commensal and pathogenic strains or isolates from healthy vs hospitalized individuals. Thomas et al. found that clusters of STs (which they call genetic clusters), rather than MLST, better explained various lifestyles in clinical settings (11). In a second study, machine-learning models were developed using genetic clusters (13). These models could distinguish between hospital and nonhospital sources with 80% accuracy, and infection vs contaminant sources with 45% accuracy (13). In a third study, Sharma et al. built machine-learning models (CART, SVM, and naïve nearest neighbor) to distinguish between blood isolates (bacteremia), contaminant isolates, and commensal isolates (skin) using MLST as features (12). The study reported CART and SVM accuracies of 73%, and 53% for KNN, concluding that the sole use of MLSTs as features was not effective as a diagnostic tool (12).

In contrast to these prior works, our study incorporated all genes, not just housekeeping genes. But rather than considering the sequences themselves, strains are represented by the presence/absence of gene homologs (common gene IDs). Moreover, all genomes considered were from healthy individuals. Both core genome analysis, which quantified genomic distance via sequence comparison, and accessory genome analysis highlight the similarity in genomic content between strains isolated from different body sites (Fig. 2 and 3, respectively). In several cases, strains from nasal, skin, and even urine samples clustered tightly together within individuals (Fig. 2; Table S2). This early signal suggested that meaningful site-specific clustering was unlikely to emerge. Therefore, we were not surprised to see a lower model accuracy.

While prior research has identified STs that are enriched in disease isolates (53–55), consistent with the observed success of machine-learning approaches based on MLST in distinguishing pathogenic from non-pathogenic strains (11–13), we did not find any association between ST and isolation source in asymptomatic females. Our model’s accuracy highlights the “generalism” of *S. epidermidis* of the human microbiota. Rather than focusing solely on predictive performance, our study provides an interpretable model linking the presence/absence of specific genes to the odds of being isolated from a specific anatomical site. While prior research has found evidence for niche adaptation among skin populations (4), we did not identify a strong singular signal of niche-specific adaptation in the sampled population. This also concurs with prior machine-learning studies that found wide variation expected of generalists and recombination between hospital-associated and nonhospital *S. epidermidis* populations (13). Our genome analysis highlights the genetic diversity of *S. epidermidis* between distal sites of the healthy human microbiota.

## MATERIALS AND METHODS

### Sample collection

Oral, skin, nasal, and urine samples were collected as part of an IRB-approved study (Loyola University Chicago, #3603); written consent was obtained from all participants. Biological females were enrolled in the study if they were between the ages of 18 and 25, self-reported as being “healthy” (asymptomatic), and self-reported that they had not taken antibiotics in the past 6 months. Oral, skin, and nasal samples were taken using swabs (BD BBL Culture Swab) after instruction and under the supervision of a researcher. The oral cavity (specifically the inside of the cheek), skin (specifically the forehead), and nose (specifically the lower inside of the nostril) were each swabbed by the participant and each swab was immediately submerged into the swab’s storage solution (Amies media). Urine samples were collected at home by participants in a supplied sterile urine collection cup. Participants were instructed on how to collect these samples, and they were asked to collect their first morning urination and bring in the sample the same day as collected.

### Bacterial isolation

Samples were processed within 1 h of receipt. Swabs were swirled and squeezed in storage media before the solution was spread on Mannitol Salt Phenol Red (MS) Agar (Millipore). For skin and urine samples, 100 µL of the solution was plated, whereas oral and nasal samples were diluted to 10^−2^ to prevent overgrowth on the plates. Dilutions were prepared using sterile 0.5% saline solution. All plates were spread following aseptic practices using cell spreaders and incubated at 35°C with 5% CO_2_ for 24 h. Each distinct colony morphology was then picked and grown in MS broth and incubated under the same culture conditions. This process was repeated to purify the strain.

### DNA extraction and sequencing

Isolates routinely producing pink or red colonies, indicative of *S. epidermidis*, were extracted using the Qiagen Blood and Tissue kit following the manufacturer’s protocol for Gram-positive organisms. DNA library preparation and sequencing were conducted by SeqCenter (Pittsburgh, PA USA) or SeqCoast (Portsmouth, NH USA). SeqCenter libraries were prepared using the tagmentation-based and PCR-based Illumina DNA prep kit and custom IDT 10 bp unique dual indices (UDI) with a target insert size of 280 bp. Libraries were then sequenced on the Illumina NovaSeqXPlus sequencer producing 2 × 151 bp paired-end reads. Demultiplexing, quality control, and adapter trimming was performed with bcl-convert1 (Illumina; v4.2.4) prior to delivery. SeqCoast libraries were prepared using Illumina’s tagmentation kit using unique dual indexes. Sequencing was performed on the Illumina NextSeq2000 sequencer producing 2 × 150 bp paired reads. Demultiplexing, read trimming, and analytics were performed with DRAGEN v4.2.7 and the on-board analysis software on the NextSeq2000.

### Genome assembly

The Bacterial and Viral Bioinformatics Resource Center (BV-BRC) webtool v3.45.4 was used to assemble the genomes with the “auto” option (56). As part of this pipeline, Trim Galore v0.6.5 (https://github.com/FelixKrueger/TrimGalore) and Unicycler v0.4.8 (57) performed read trimming and assembly, respectively. Assemblies were refined using Pilon v1.23 (58). Genomic binning was conducted on all assemblies with the RASTtk v1.073 pipeline (59) producing bins assigned to taxa. The process of read binning identified some samples consisting of discrete bins representative of different species, indicating that the isolate was not purified prior to DNA extraction. Genomes and metagenomic assembled genomes (MAGs) were identified as *S. epidermidis* by BV-BRC’s metagenomic binning function. The study described here only includes genomes with a sequence completeness above 97% and contamination below 5%, computed by BV-BRC using the tool CheckM v1.2.3 (60). Genome annotation was conducted using the NCBI Prokaryotic Genome Annotation Pipeline (PGAP) v6.7 (61), and all samples are placed under BioProject PRJNA1046043. Details regarding *S. epidermidis* genomes produced from these samples included in Table S1.

### MLST analysis

Following MLST assignment using the PubMLST allele database (62), all sequence types (STs) and allele calls were merged with sample metadata describing the anatomical site of origin for each of the 114 isolates.

### Genome comparisons

The pangenome was constructed for the participant-derived genomes using Anvi’o v8 (63). All assembled genomes were converted to Anvi’o format using the anvi-gen-contigs command. Gene calling was performed using Prodigal v2.6.3 (64), the default gene prediction algorithm integrated into Anvi’o. The pangenome was constructed using the anvi-pan-genome command with a Markov Cluster Algorithm (MCL) parameter value of 10. This MCL threshold was selected because all genomes being compared are representatives of the same species. Per the Anvi’o workflow for microbial pangenomics (https://merenlab.org/2016/11/08/pangenomics-v2/; last modified on November 08, 2016), an MCL of 10 is recommended for comparing strains of the same species. Single copy number genes present in all 114 genomes were identified using the anvi-get-sequences-for-gene-clusters command with the following parameters: “--min-num-genomes-gene-clusters-occurs 114 --max-num-genes-from-each-genome 1 --concatenate-gene-clusters.” A phylogenetic tree was derived using FastTree v2.1.11 (65), through Geneious Prime 2024.0.7 (GraphPad Software, L.L.C.), with default parameters. The resulting Newick format file was output and then visualized in iTOL v7.0, with circular mode representation (66).

Pairwise ANI values using FastANI v1.34 (67) through Anvi’o. The full ANI matrix was refined to include just those from participants in which genomes were produced from more than one isolation site and this matrix was visualized using Python’s matplotlib library.

The accessory genome was further examined according to the functionality encoded. Using Anvi’o annotations, the COG20 functions for the genomes were exported as a presence/absence matrix using the anvi-script-gen-function-matrix-across-genomes command. A Jaccard dissimilarity matrix was computed on this binary data and used to perform Principal Coordinates Analysis (PCoA) in R. An FDR analysis was performed to determine if any particular COG was enriched for a particular isolation source. The code used for this analysis is publicly available at: https://github.com/nshanbhag123/Strain-Level-Genomic-Analysis-of-S.-epidermidis.

### Prophage analysis

Prophage sequences within the participant-derived genomes were identified using the webtool PHASTEST (68) via its API functionality, with default parameters. Phage categories—intact, questionable, and incomplete—were parsed from the results, with only intact phages included in subsequent analysis. Identified intact prophages were analyzed using ViPTree v4.0 (69), with the tool’s references. The resulting tree was visualized to include the user-supplied sequences and related genomes as a circular tree. To quantitatively assess intergenomic relatedness of the predicted intact prophages, the VIRIDIC (Virus Intergenomic Distance Calculator) webserver (70) was used. VIRIDIC thresholds of 95% and 70% were applied to delineate species- and genus-level similarity, respectively, and a heatmap of pairwise comparisons was generated by the tool. Details about the intact prophage sequences examined can be found in Table S3.

### ARG identification

All participant-derived genomes were analyzed using the Comprehensive Antibiotic Resistance Database (CARD) v3.1.4 (71) to identify the presence of antibiotic resistance genes (ARGs). ARGs were predicted using the Resistance Gene Identifier (RGI), employing the BLAST algorithm, to detect resistance determinants. Only perfect and strict matches were considered further, while loose hits—which indicate lower-confidence predictions—were excluded.

### Virulence factor detection

Virulence factors were identified through comparing each of the 114 *S. epidermidis* genomes against the Virulence Factor Database (VFDB) (72). The combined VFDB core and full data sets were downloaded and formatted as a local BLAST reference database. Participant genomes and reference genes from VFDB were compared to each other using BLASTx with default parameters, and hits were filtered using the following thresholds: a minimum of 80% percent sequence identity and 80% query coverage.

### Machine learning and gene clusters

Publicly available *S. epidermidis* genomes (*n* = 108) were retrieved from NCBI’s Genome database, selected based upon the site of isolation per the genome record’s metadata (Table S5). For nasal and skin isolates, genomes were randomly chosen from those publicly available with metadata indicating that they were from healthy individuals. For urine isolates, selection was based on inclusion criteria requiring the genome to be from an asymptomatic female host. These publicly available genomes were subject to the same quality control as the genomes produced in this study; genome completeness (>97%) and contamination (5%) were evaluated using CheckM v1.2.3. Species assignment for these genomes was further verified using the JSpecies Web Server (73). In total, we had 33 genomes of skin isolates, 38 genomes of nasal isolates, and 37 genomes from urinary isolates. These genomes were added to the participant-derived genome set produced here and a pangenome was created using Anvi’o as previously described. The pangenome gene presence/absence matrix was output for use in our modeling.

To classify *S. epidermidis* genomes based on their isolation source, we developed a multinomial logistic regression (MLR) model using gene cluster presence/absence data. All analyses were performed in Python, using the following packages: pandas for data handling, numpy for numerical operations, scikit-learn for model training and feature selection, and matplotlib and seaborn for visualization. The code used for this analysis is publicly available at: https://github.com/nshanbhag123/Strain-Level-Genomic-Analysis-of-S.-epidermidis.

The 108 public genomes presence/absence matrix was used exclusively as the testing set, while the 104 participant-derived presence/absence matrix (after removing oral samples) were used as the training set. An “Isolation_Source” was numerically encoded using LabelEncoder (74). Next, a machine-learning pipeline was created to ensure reproducibility and prevent data leakage between steps. The first part of the pipeline included application of a variance threshold filter using the VarianceThreshold package from the scikit-learn library (74). Gene clusters with a variance below a certain threshold across the training samples were excluded prior to model fitting, as they lacked the variability necessary to distinguish between classes. The second part of the pipeline included fitting a multinomial logistic regression model using a specific regularization parameter C for L2 regularization (also known as ridge regression). The model employed the saga solver (75), which is well-suited for sparse, high-dimensional data and supports multiclass classification. Additionally, the model was allowed up to 10,000 iterations to ensure convergence. Since the optimal variance threshold and regularization parameter C were unknown, we employed GridSearchCV (74). For the grid, we tested the following variance thresholds: 0.05, 0.10, 0.15, 0.20. The regularization parameter C was selected from a logarithmically spaced range of 10 values between 1e-4 and 1e4. In total, the grid consisted of 40 hyper-parameter combinations. The combinations were evaluated through fivefold stratified cross-validation, a robust and reliable way to measure model performance, by utilizing five different training and testing sets. We chose to utilize a scoring metric of negative log loss, as this metric not only penalizes misclassification, but the confidence of misclassification. Our model found that a variance threshold of 0.20 and a regularization parameter C of 0.006 were associated with the lowest average loss across 5-fold, and 115 iterations were needed for convergence of this model.

This machine-learning pipeline, using the optimal hyper-parameters, was then used to predict the isolation sites from the publicly available testing data. To interpret the results and identify the gene clusters most strongly associated with each isolation source, we extracted the coefficient matrix from the trained logistic regression model. To focus the interpretation on the most relevant features, we retained only those gene clusters with an absolute coefficient value greater than 0.025 in at least one class. The retained gene clusters we refer to as key gene clusters. The filtered coefficients were visualized using a cluster map, generated using Seaborn (76).

To further contextualize the top-ranked features identified by the model, gene cluster identifiers were mapped to functional annotations by cross-referencing them with a gene cluster summary file generated by Anvi’o, which included COG20 categories and associated functional descriptions. Mapping was performed by matching gene cluster identifiers to their corresponding COG annotations within the summary file. If one gene cluster mapped to a known COG function and an unknown COG function, the known COG function was reported. If a gene cluster was not mapped to a known COG function via Anvi’o, the amino acid sequences were manually annotated for function using UniProt (v.2025) (77) and NCBI database searches (78), and the COG function associated with best coverage and homologous similarity was reported. The final curated table, linking each top-ranked gene cluster to its predicted biological function, was exported as a CSV file with corresponding COG (Cluster of Orthologous Groups) functions, as annotated by Anvi’o, or by UniProt.

## SUPPLEMENTAL MATERIAL

Fig. S1 spectrum.00114-26-s0001.pdf Presence and absence of *S. epidermidis* isolates from four anatomical sites.

Fig. S2 spectrum.00114-26-s0002.pdf Proteomic tree of intact prophages.

Table S1 spectrum.00114-26-s0003.xlsx Genome information.

Table S2 spectrum.00114-26-s0004.xlsx ANI matrix.

Table S3 spectrum.00114-26-s0005.xlsx Prophage taxa.

Table S4 spectrum.00114-26-s0006.xlsx Genomes containing ARGs by isolation source.

Table S5 spectrum.00114-26-s0007.xlsx Publicly available genomes used in our model test data set.

ASM does not own the copyrights to Supplemental Material that may be linked to, or accessed through, an article. The authors have granted ASM a non-exclusive, world-wide license to publish the Supplemental Material files. Please contact the corresponding author directly for reuse.
