# Supplementary material for: Strain-level genomic analysis of Staphylococcus epidermidis across multiple body sites in healthy females
Source: Microbiol Spectr. 2026 Jun 22;14(8):e00114-26. doi: 10.1128/spectrum.00114-26 (PMC13436424; doi:10.1128/spectrum.00114-26)
Supplement: Supplementary file 1 [file spectrum.00114-26-s0001.pdf]

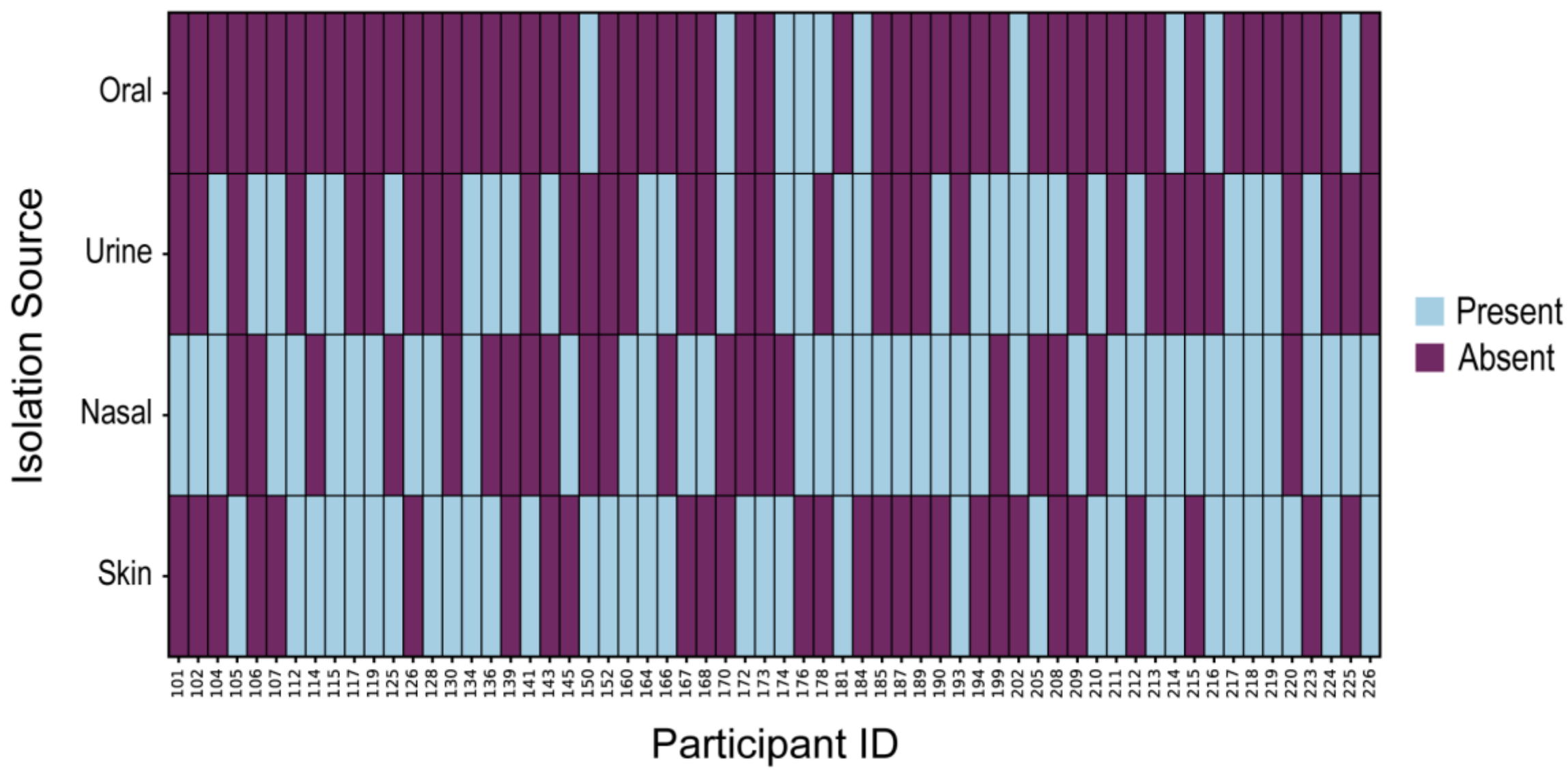

Figure S1. Presence and absence of *S. epidermidis* isolates from four anatomical sites (skin, nasal, urine, oral) across study participants. Each square indicates whether *S. epidermidis* was successfully isolated from a given site for each participant. Purple represents absence and light blue indicates presence. Participant IDs are ordered numerically along the x-axis.
