# Supplementary material for: Strain-level genomic analysis of Staphylococcus epidermidis across multiple body sites in healthy females
Source: Microbiol Spectr. 2026 Jun 22;14(8):e00114-26. doi: 10.1128/spectrum.00114-26 (PMC13436424; doi:10.1128/spectrum.00114-26)
Supplement: Supplementary file 2 [file spectrum.00114-26-s0002.pdf]

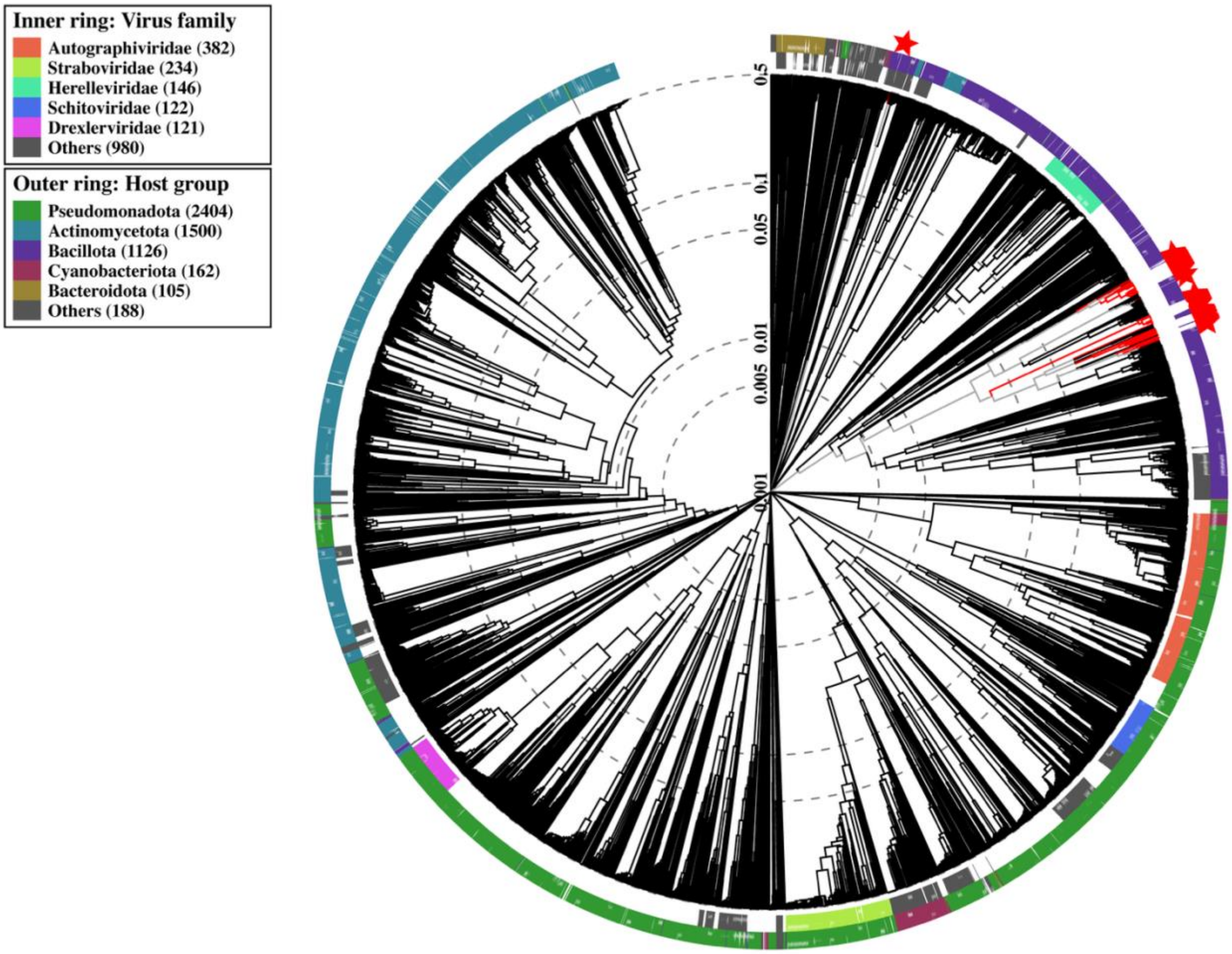

Figure S2. Proteomic tree generated by ViPTree, displaying all viral sequences identified as intact prophages via PHASTEST, along with reference phage genomes from the ViPTree database. Red stars and branches indicate prophage sequences identified in *S. epidermidis* genomes collected in this study.
